# Clinician Preimplementation Perspectives of a Decision-Support Tool for the Prediction of Cardiac Arrhythmia Based on Machine Learning: Near-Live Feasibility and Qualitative Study

**DOI:** 10.2196/26964

**Published:** 2021-11-26

**Authors:** Stina Matthiesen, Søren Zöga Diederichsen, Mikkel Klitzing Hartmann Hansen, Christina Villumsen, Mats Christian Højbjerg Lassen, Peter Karl Jacobsen, Niels Risum, Bo Gregers Winkel, Berit T Philbert, Jesper Hastrup Svendsen, Tariq Osman Andersen

**Affiliations:** 1 Department of Computer Science Faculty of Science University of Copenhagen Copenhagen Denmark; 2 Vital Beats Copenhagen Denmark; 3 Department of Cardiology Rigshospitalet Copenhagen University Hospital Copenhagen Denmark; 4 Department of Clinical Medicine Faculty of Health and Medical Sciences University of Copenhagen Copenhagen Denmark

**Keywords:** cardiac arrhythmia, short-term prediction, clinical decision support systems, machine learning, artificial intelligence, preimplementation, qualitative study, implantable cardioverter defibrillator, remote follow-up, sociotechnical

## Abstract

**Background:**

Artificial intelligence (AI), such as machine learning (ML), shows great promise for improving clinical decision-making in cardiac diseases by outperforming statistical-based models. However, few AI-based tools have been implemented in cardiology clinics because of the sociotechnical challenges during transitioning from algorithm development to real-world implementation.

**Objective:**

This study explored how an ML-based tool for predicting ventricular tachycardia and ventricular fibrillation (VT/VF) could support clinical decision-making in the remote monitoring of patients with an implantable cardioverter defibrillator (ICD).

**Methods:**

Seven experienced electrophysiologists participated in a near-live feasibility and qualitative study, which included walkthroughs of 5 blinded retrospective patient cases, use of the prediction tool, and questionnaires and interview questions. All sessions were video recorded, and sessions evaluating the prediction tool were transcribed verbatim. Data were analyzed through an inductive qualitative approach based on grounded theory.

**Results:**

The prediction tool was found to have potential for supporting decision-making in ICD remote monitoring by providing reassurance, increasing confidence, acting as a second opinion, reducing information search time, and enabling delegation of decisions to nurses and technicians. However, the prediction tool did not lead to changes in clinical action and was found less useful in cases where the quality of data was poor or when VT/VF predictions were found to be irrelevant for evaluating the patient.

**Conclusions:**

When transitioning from AI development to testing its feasibility for clinical implementation, we need to consider the following: expectations must be aligned with the intended use of AI; trust in the prediction tool is likely to emerge from real-world use; and AI accuracy is relational and dependent on available information and local workflows. Addressing the sociotechnical gap between the development and implementation of clinical decision-support tools based on ML in cardiac care is essential for succeeding with adoption. It is suggested to include clinical end-users, clinical contexts, and workflows throughout the overall iterative approach to design, development, and implementation.

## Introduction

Ventricular tachycardia and ventricular fibrillation (VT/VF) are potentially lethal cardiac arrhythmias, which constitute a growing challenge to health care systems worldwide [1]. The development of implantable cardioverter defibrillators (ICDs) has led to major advances in the prevention of death from VT/VF [2]. ICDs are implantable devices used in patients at increased risk of sudden cardiac death. ICDs monitor the heart rhythm continuously to detect and treat VT/VF. In recent years, remote monitoring has become the standard of care for ICD patients [3], and follow-ups are based on transmission of data from the implanted device through the patient’s home monitoring box. This has reduced the number of in-office follow-ups [4,5] and increased survival rates [6] due to improved early detection of arrhythmias [7]. However, the numbers of ICD implants are increasing worldwide, posing a workload challenge for electrophysiologists and technicians when assessing data from incoming transmissions in remote monitoring centers [8-11]. There is a growing need for decision-making tools that can support and reduce data-intensive remote follow-ups, and while current systems can detect and treat VT/VF arrhythmias as they occur, tools for predicting arrhythmias before their onset are lacking [12].

Artificial intelligence (AI), such as machine learning (ML), shows great promise for improving clinical decision-making in cardiac diseases by outperforming statistical-based models [12,13], and recent examples include promising models for the prediction of heart disease and heart failure [14-18], as well as cardiac arrhythmias, such as ventricular arrhythmia [19], atrial fibrillation [20], and electrical storm [21]. There are positive attitudes and high expectations among physicians that AI will improve future patient care in fields where data are collected continuously, such as cardiology [22,23].

However, few prediction outcome algorithms based on ML have been implemented in cardiology clinics because of the challenges during transitioning from algorithm development to real-world implementation. While studies of medical AI-based tools that undergo prospective clinical validation are emerging [24-27], there is a general lack of understanding of how AI may support achieving clinical effectiveness and improve patient care in real-life settings [28,29]. Scholars have argued that ML-based patient outcome prediction models are yet to prove their worth to human clinicians [30]. Prediction accuracy by itself can be impressive in the lab; however, this does not always translate to better treatment, and it is being stressed to look for ways to make human and AI prediction algorithms complement each other, ensuring actionability in clinical practice [30-33]. Going from research and development environments to hospital or clinical contexts is considered a challenging task that has been named “the last mile” of implementing medical AI-based tools [34,35], and there is a call for research on how end-users find AI-based user interfaces useful in practice [36-39], as well as studies that report on the sociotechnical challenges of deploying AI-based tools in complex clinical environments [27,34,35,40-54].

This study addresses the sociotechnical gap between the development and implementation of a clinical decision-support tool based on ML for the prediction of VT/VF in remote monitoring of ICD patients. The aim of this study was to explore the feasibility and clinician preimplementation perspectives of using a prediction tool for improved workflows. Therefore, this study does not provide algorithmic validation per se but instead answers questions about the clinical feasibility and workflow integration of a decision-support tool based on ML.

## Methods

### Understanding Needs and Co-design of the Prediction Tool

**Figure 1:**
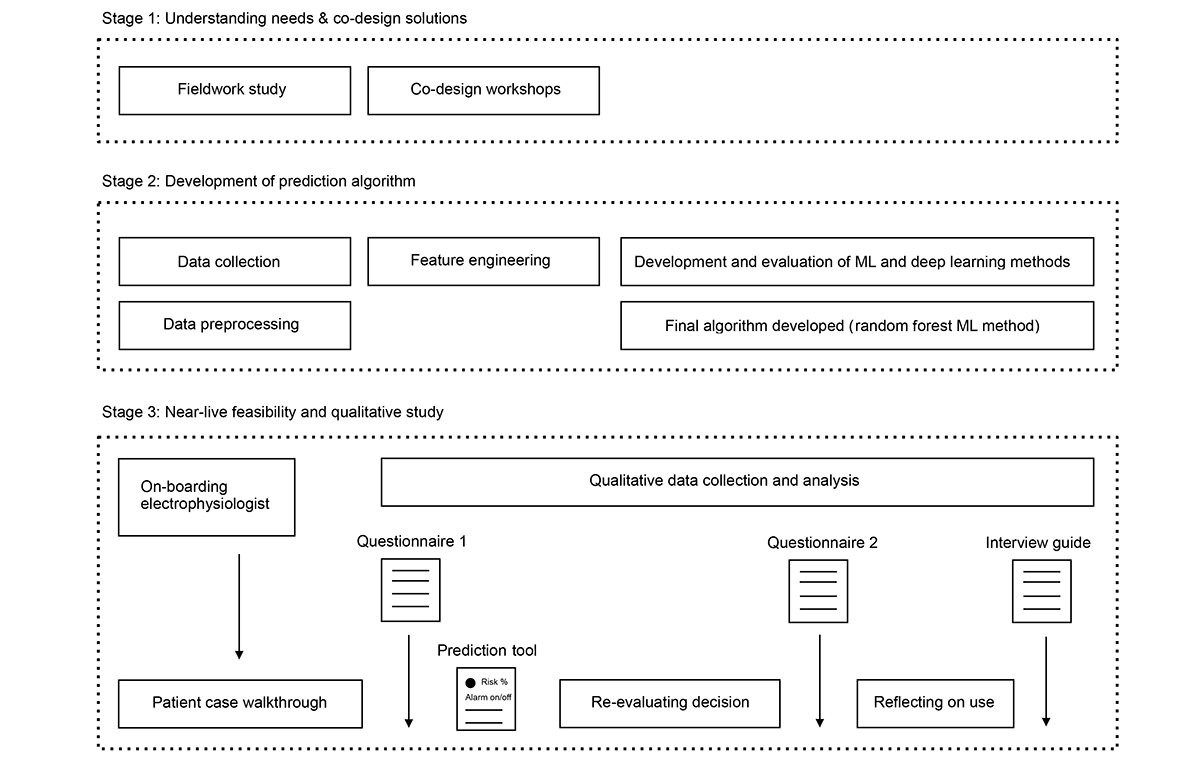
Overall study design. ML: machine learning.

This study was conducted at the remote monitoring center at Rigshospitalet, Copenhagen University Hospital, Denmark, which is a large tertiary hospital covering all aspects of treatments in cardiology and is among the largest centers in Europe having more than 4000 patients with cardiac implanted electronic devices in remote follow-up. The study was organized in 3 stages (Figure 1). In the first stage, field work observations in the remote monitoring clinic were conducted to understand both the clinical workflow and workload [10]. This was followed by 3 co-design workshops with an electrophysiologist (PKJ) and 5 co-design workshops with a cardiologist consultant (SZD) focusing on feature engineering and sketching the user interface. In stage 2, the AI algorithm was developed, and in stage 3, a near-live feasibility and qualitative interview study was conducted. The study was reviewed by the Danish National Board of Health and the Danish National Committee on Health Research Ethics, and authorized by The Capital Region of Denmark.

### Development of the AI Algorithm

**Figure 2:**
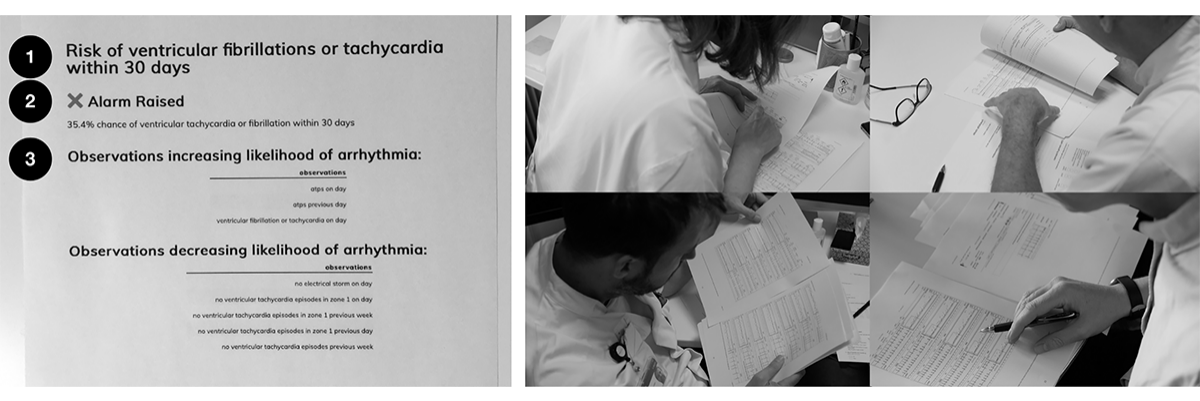
The prediction tool on a paper printout as shown to study participants (Case 3, see Table 2). The output shows the alarm (yes/no), risk probability (%), and up to 5 most important parameters for increasing and decreasing the likelihood of ventricular tachycardia and ventricular fibrillation within 30 days. To the right: example pictures of electrophysiologists conducting near-live case walkthroughs.

A prediction tool was developed for improving the support for clinical decision-making in ICD remote monitoring based on the random forest ML method, and it consisted of a risk prediction algorithm of VT/VF within 30 days. The prediction tool was designed to show alarm status (yes/no), risk probability (%), and ranking of the 5 most and least important parameters for the prediction, using the LIME technique [55] (Figure 2). The design and development of the tool were informed by previous fieldwork studies of current practices [10,56], as well as early results from using ML to predict electrical storm, a severe form of cardiac arrhythmia [21]. The data set used for developing the algorithm consisted of 11,921 transmissions from 1251 patients with an ICD or a cardiac resynchronization therapy defibrillator (CRT-D), followed over a 4-year period from 2015 to 2019 at Rigshospitalet. The data set contained 74,149 arrhythmia episodes, each characterized by 7 variables, such as the type of arrhythmia (VT, VF, supraventricular tachycardia, atrial fibrillation, etc), ICD treatment of the arrhythmia, duration of the episode, and maximum heart rate reached during the episode.

The random forest ML method [44] was selected for algorithm development because it provided optimal results when considering the tradeoffs between model performance and explainability. Several other classifier methods (supervised, unsupervised, and deep learning methods) were evaluated through development and testing, including KNeighborsClassifier [57], GradientBoostingClassifier [58], AdaBoostClassifier [59], support vector classifier [60], and long short-term memory (LSTM) [61]. The deep learning method, LSTM, provided poorer performance and poorer explainability, possibly due to the nature of the data (ie, time series data with considerable time between events, making time series modeling difficult). The other methods provided similar performance. KNeighborsClassifier and support vector machine had the worst performance, while the decision tree methods had the best performance. GradientBoostingClassifier produced an optimal F1 score and recall score; however, random forest provided the highest accuracy and precision scores, which led to the choice of using the random forest method for developing the first version of the algorithm to be evaluated with end-users in this study. The algorithm was tested on 2342 of the 11,921 transmissions. The transmission data were stratified and grouped into training and test sets. This means that the prevalence of the positive condition was the same in both the training and test sets (stratified) and that no patient had data in both data sets (grouped). The algorithm achieved an accuracy of 0.96, with a positive predictive value of 0.67 and a negative predictive value of 0.97. The probability threshold for raising an alarm was set to 0.28, indicating the value with an optimal tradeoff between negative and positive predictive outcomes.

Feature engineering was carried out in collaboration between 2 data scientists (MKHH and CV) and a cardiologist consultant (SZD) during 5 co-design workshops. A total of 48 features (referred to as parameters when discussed with the study participants) were developed, and the following 2 main principles were adopted: aggregating episodes by day and building a historic snapshot for days leading up to the arrhythmic event. To provide the clinical end-user with algorithm explainability, the LIME technique [55] was used to show the top 5 features that increase or decrease the likelihood of a VT/VF arrhythmic event occurring within the coming 30 days.

### Study Participants and Case Selection

Seven medical doctors specialized in electrophysiology (ie, cardiologists treating patients with cardiac arrhythmia) were selected for participation from a convenience sample (Table 1). Participants included 6 males and 1 female (average age, 52 years; average work experience as an electrophysiologist, 13 years).

A selection of 5 retrospective patient cases (Table 2) was used to evaluate the feasibility of the prediction tool’s ability to support clinical decision-making. The cases included high and low risk probability, true positives, and true negatives, and 2 cases with AF as the primary episode type. Patient cases were retrieved 19 to 27 months back in time, blinded, and presented as paper printouts with a summary of each patient’s clinical history along with reports from the electronic health record (list of diagnoses, progress notes from the cardiology department, latest blood tests, and list of medications) and screenshots of relevant ICD transmission data, including device type, battery status, device programming and settings, time of implantation, latest diagnostic information about the transmission, frequency of arrhythmias, heart rate, device therapy, and assessment of physical activity.

**Table 1:** Participating electrophysiologists.

Participant	Sex	Age (years)	Title	Years since obtaining specialist certification in cardiology
1	Female	52	Consultant cardiologist, MD, PhD	11
2	Male	61	Professor, consultant cardiologist, MD, DMSc	23
3	Male	55	Consultant cardiologist, MD, PhD	14
4	Male	43	Cardiologist, MD, PhD	2
5	Male	62	Consultant cardiologist, MD, DMSc	28
6	Male	44	Cardiologist, MD, PhD	2
7	Male	47	Consultant cardiologist, MD, DMSc	9

**Table 2:** Case overview with patient summary, current implantable cardioverter defibrillator transmission information, and prediction tool information.

Case number	Patient summary	Current ICD^a^ transmission					Prediction tool	
		Transmission type	Primary episode type	ICD treatment	Transmission summary	30-day VT^b^/VF^c^ risk probability		Alarm raised (prediction outcome)
1	Male, age 63 years, ischemic heart failure, left ventricular assist device	Automated	VT/VF	ATP^d^	3 VT/VF; 36 sensing episodes; 217 VT-NS^e^	58.6		Yes (true positive)
2	Female, age 67 years, dilated cardiomyopathy	Automated	VT/VF	Shock	1 VT/VF; 1 VT-NS; 20 min of AF^f^ since the last transmission	14.4		No (true negative)
3	Female, age 40 years, dilated cardiomyopathy	Automated	VT/VF	Shock	2 VT/VF; 4 VT-NS	35.4		Yes (true positive)
4	Male, age 61 years, ischemic heart failure	Patient initiated	AF	None	12 hours of AF since the last transmission	1.2		No (true negative)
5	Male, age 73 years, ischemic heart failure	Automated	AF	None	14 hours of AF since the last session; 26 VT-NS	7.8		No (true negative)

^a^ICD: implantable cardioverter defibrillator.

^b^VT: ventricular tachycardia.

^c^VF: ventricular fibrillation.

^d^ATP: antitachycardia pacing.

^e^VT-NS: nonsustained ventricular tachycardia.

^f^AF: atrial fibrillation.

### Data Collection

A combined feasibility and qualitative interview study was undertaken based on a retrospective case study design. The primary aim of the study was to address the following 4 main questions about the feasibility of the prediction tool using quantitative measures: Does use of the tool lead to change in clinical action? Does it support decision-making? Are visualizing parameters useful? Can it reduce time spent? The secondary aims were to understand the electrophysiologist’s immediate reactions to using the prediction tool, including qualifying the quantitative feasibility measures against qualitative dimensions based on interviews. Electrophysiologists were invited to conduct a “near-live” clinical simulation of decision-making based on walkthroughs of the 5 patient cases (Table 2) with and without the prediction tool. Two structured questionnaires based on a 5-point Likert scale were designed to capture electrophysiologists’ decisions on action without the prediction tool (Multimedia Appendix 1) and their experiences of the feasibility of the prediction tool (Multimedia Appendix 2). A semistructured interview guide was designed based on the framework of Bowen et al for feasibility studies [62] to cover open-ended questions about the electrophysiologists’ overall experiences of using the prediction tool. Ten questions in the following 4 areas of inquiry were posed: acceptability, demand, adoption, and implementation (Multimedia Appendix 3).

“Near-live” case walkthroughs were performed with inspiration from Li et al [63] (Figure 1), and they were facilitated by the authors SM, MKHH, and TOA. First, the electrophysiologist was on-boarded with a presentation of the study objectives, the intended use of the prediction tool, the algorithm development (data set and ML model, as well as results), and the outline of the feasibility and qualitative study processes, and time was provided to resolve open questions. Second, the electrophysiologist was provided with a patient case and asked to do a walkthrough of the case material to reach a decision on clinical action, similar to normal clinical practice, and was asked to answer the first questionnaire and explain the reasoning behind the decision on clinical action. Third, the electrophysiologist received the prediction tool on paper and was asked to answer the second questionnaire for evaluation of the effects of the prediction tool and to share his/her immediate reactions. Fourth, after ending all patient case walkthroughs, the electrophysiologist was interviewed about his/her experience of the feasibility of the prediction tool. The total time for observations and interviews was 12.5 hours, with an average of 1 hour 47 minutes per electrophysiologist. Case walkthroughs and interviews were audio and video recorded, and sections with electrophysiologists’ responses to the questionnaires and the open-ended interview were transcribed verbatim.

### Data Analysis

Data from electrophysiologists’ reactions to the interview study were analyzed using an inductive qualitative approach based on grounded theory [64]. A 2-step iterative coding process was applied beginning with line-by-line coding to support initial analytic decisions about the data. Action codes were developed by using gerunds (a noun form of a verb) to make explicit what electrophysiologists were doing during case walkthroughs and what meaning they derived (eg, “being confirmed,” “building trust,” and “using to prioritize”). This was done to preserve focus on action and situated processes of electrophysiologists’ decision-making, and to turn thematic descriptions into analytical insights in later stages of the analysis. Focused coding was carried out by iteratively sorting and synthesizing line-by-line codes into themes and subthemes related to the research questions and by constructing key insights. This process allowed for comparing and turning frequently reappearing initial codes across large amounts of data, and obtaining more general and analytically incisive findings (eg, “predictions can serve as a second opinion” and “decision-making workload is reduced when trust in the prediction tool is established”). The entire process was carried out iteratively in collaboration between SM and TOA using the qualitative data analysis software NVivo 12 (QSR International).

## Results

### Feasibility of the Prediction Tool in Clinical Practice

#### Does the Prediction Tool Change Clinical Decisions?

Overall, the electrophysiologists did not change their decisions on clinical action when presented with the 30-day VT/VF arrhythmia prediction (Table 3). However, several electrophysiologists found that the prediction tool was helpful (Textbox 1, Quote 1) and increased their confidence in their choice of clinical action, and that the predictions could help prioritize patients (Textbox 1, Quote 2) and determine what action to take in relation to the local circumstances at the clinic (Textbox 1, Quote 3).

**Table 3:** Effect of the prediction tool on electrophysiologists’ decision-making.

Question and answer		Total (N=35), n (%)	Case 1 (N=7), n (%)	Case 2 (N=7), n (%)	Case 3 (N=7), n (%)	Case 4 (N=7), n (%)	Case 5 (N=7), n (%)
**Q1: The prediction tool made me change my decision on clinical action**							
	Yes	1 (3)	1 (14)	0 (0)	0 (0)	0 (0)	0 (0)
	No	34 (97)	6 (86)	7 (100)	7 (100)	7 (100)	7 (100)
**Q1a: I will contact the patient**							
	Strongly disagree/disagree	0 (0)	0 (0)	0 (0)	0 (0)	0 (0)	0 (0)
	Neither agree nor disagree	0 (0)	0 (0)	0 (0)	0 (0)	0 (0)	0 (0)
	Agree/strongly agree	35 (100)	7 (100)	7 (100)	7 (100)	7 (100)	7 (100)
**Q1b: I will book a procedure or reschedule an existing procedure**							
	Strongly disagree/disagree	3 (9)	0 (0)	1 (14)	1 (14)	0 (0)	1 (14)
	Neither agree nor disagree	6 (17)	1 (14)	0 (0)	0 (0)	4 (57)	1 (14)
	Agree/strongly agree	26 (74)	6 (86)	6 (86)	6 (86)	3 (43)	5 (71)
**Q1c: I will do something else**							
	Strongly disagree/disagree	19 (54)	5 (71)	3 (43)	5 (71)	2 (29)	4 (57)
	Neither agree nor disagree	5 (14)	1 (14)	1 (14)	1 (14)	2 (29)	0 (0)
	Agree/strongly agree	11 (31)	1 (14)	3 (43)	1 (14)	3 (43)	3 (43)
**Q2: The prediction tool supported my decision-making**							
	Strongly disagree/disagree	8 (23)	2 (29)	1 (14)	0 (0)	3 (43)	2 (29)
	Neither agree nor disagree	4 (11)	1 (14)	0 (0)	1 (14)	1 (14)	1 (14)
	Agree/strongly agree	23 (66)	4 (57)	6 (86)	6 (86)	3 (43)	4 (57)
**Q3: The prediction tool’s visualization of parameters supported my decision making**							
	Strongly disagree/disagree	11 (31)	3 (43)	1 (14)	2 (29)	3 (43)	2 (29)
	Neither agree nor disagree	3 (9)	1 (14)	0 (0)	0 (0)	1 (14)	1 (14)
	Agree/strongly agree	21 (60)	3 (43)	6 (86)	5 (71)	3 (43)	4 (57)
**Q4: The prediction tool can help me reach a decision faster**							
	Strongly disagree/disagree	13 (37)	4 (57)	2 (29)	2 (29)	2 (29)	3 (43)
	Neither agree nor disagree	4 (11)	1 (14)	0 (0)	0 (0)	3 (43)	0 (0)
	Agree/strongly agree	18 (51)	2 (29)	5 (71)	5 (71)	2 (29)	4 (57)

**Themes, insights, and illustrative quotes describing the feasibility of the prediction tool.**

**Taking Action**

**Insights:** The prediction tool led to no change in clinical action; the prediction tool can increase confidence in clinical action; the prediction tool can help prioritize clinical action and patients; and being confirmed supports decision-making.

- **Quote 1:*** Well, it hasn’t changed my current decision, but the basis is much better, and I can easily see that it has helped me.* [Case 3, Electrophysiologist #7]
- **Quote 2:*** If you are in a busy situation where many transmissions have arrived and the technician and I have to maneuver and prioritize, there is no doubt that we will concentrate on those with high-risk predictions.* [Electrophysiologist #5]
- **Quote 3:*** This [tool prediction] is something that might make me react a little more aggressively. […] Now I've been told that he's actually more likely to get an episode within the next month than he's not getting an episode […] if our program is fully booked, both today and tomorrow, and the day after tomorrow, but on Friday we have a time. Then I kind of have to make a trade off if I really want to spare him a shock. Which may turn into a lot of shocks.* [Case 1, Electrophysiologist #2]

**Decision-Making**

**Insights:** The prediction tool predictions served as a second opinion; the prediction tool supported gathering of thoughts; the overall presentation of the prediction tool needs to be easily translatable to clinical relevance; and being confirmed supports decision-making.

- **Quote 4:*** So, I agree with the conclusion, it was also my feeling that I would be a little worried about this patient.* [Case 3, Electrophysiologist #6]
- **Quote 5:*** But then if it is you now have to convince some [other electrophysiologists] that they should ablate her, then instead of saying that I think so, you can argue that the algorithm thinks so too. So, in that way you can say that you can get an extra view of it.* [Case 3, Electrophysiologist #3]
- **Quote 6:*** In that way, the algorithm can be a support because it helps to gather thoughts about things that play a role in whether a person gets a new arrhythmia.* [Case 3, Electrophysiologist #5]
- **Quote 7:*** Yes, I think again that if you present 58.6% then it expresses an accuracy that you may have difficulty navigating with. I know it from other areas in the medical world, the thing about expressing something with a decimal number, it expresses an accuracy for which there may be no evidence at all […] I have a hard time relating to the number […] it’s problematic to translate that into something clinically relevant.* [Case 1, Electrophysiologist #5]
- **Quote 8:*** I agree with what the alarm tells me, but I don’t think it has helped me very much right here.* [Case 5, Electrophysiologist #5]

**Visualization**

**Insights:** The prediction tool should provide actionable parameters; showing parameters enables confirmation and agreement; showing parameters enables in-situ validation of algorithmic inputs and the prediction tool result; the prediction tool performs only as good as the data it bases its predictions on; transparency about the algorithmic data input helped raise confidence and trust; and showing important parameters is more important than showing the output probability.

- **Quote 9:*** To list what counts for and what counts against, makes really good sense. That’s also how it works in my head.* [Case 3, Electrophysiologist #7]
- **Quote 10:*** I think it's super good, I actually think it's really pedagogical, I like it. Because, in reality this is how it confirms the result. It’s basically the same empirical data that you have in your mind: You say “okay, is this a case where we have to do something?” It sums up some assumptions that you have made yourself, and in that way, I actually think you are confirmed more than if you have a green or red light.* [Electrophysiologist #4]
- **Quote 11:*** It's very nice to see that the algorithm reacts on the same parameters that I've discovered myself ... So it's nice to see that I agree with it. You could say that it’s supporting and it's safe to know, that it also says there was something here.* [Case 3, Electrophysiologist #1]
- **Quote 12:*** What's happening here is that the ICD detects that the patient has VT, and then the prediction tool bases it’s predictions on that. But it’s not entirely correct, because the device has recently been re-programmed to sense everything.* [Case 1, Electrophysiologist #6]
- **Quote 13:*** This case has nothing to do with risk of VT/VF [...] it's the second thing I look at. No, here I won’t use it [the prediction tool].* [Case 4, Electrophysiologist #1]

 

**Time Saving**

**Insights:** The prediction tool can speed up decision-making when trust is established; the prediction tool can reduce workload when trust is established; the prediction tool can reduce information search time when no or low risk is predicted; the prediction tool can substitute patient input; and showing important parameters enables work redelegation to technicians and nurses.

- **Quote 14:*** It will give me a much better basis for decision making and I actually think it will save me a lot of time. Just like with all other new technology based on machine learning: the first 2 months I sit and read through to see what I have, but in month 3, I will look at the output alone. Because then I trust that it has pulled out what is appropriate, and then it starts saving me all the work I did in the beginning. But for everyone, it is that there is a phase for you personally to find out if this brings you further. […] I really think I would have come to the decision faster if I had seen this first.* [Case 3, Electrophysiologist #7]
- **Quote 15:*** I might reach a decision faster with this system if I can’t get a hold of the patient i.e., if the patient does not pick up the phone. Then it could well be that I look at the alarm and say “well, yes okay there is low risk.”* [Case 2, Electrophysiologist #6]
- **Quote 16:*** You could make a scenario where the technician first looks at it [the prediction tool] and says ... okay there are those parameters and there is electrical storm, so we call in the patient and the doctor does not have to look at the transmission. That would support our workflow.* [Case 1, Electrophysiologist #4]

#### In Which Cases Does the Prediction Tool Support Clinical Decision-Making?

In 23 (66%) of the case walkthroughs, the electrophysiologists agreed that the prediction tool supported their decision-making, whereas in 8 (23%) of the walkthroughs they disagreed. Finding the prediction tool supporting was particularly pertinent in both patient cases 2 and 3, where 6 (86%) of the electrophysiologists agreed, and the prediction tool was found to assist decision-making by confirming the electrophysiologists’ clinical evaluations and expectations of an increasing risk of VF/VT (Textbox 1, Quote 4). On the contrary, when the electrophysiologists were focused on predicting arrhythmias other than VT/VF, the prediction tool was deemed less useful, and answers were more heterogenous (Case 4 and Case 5). Some electrophysiologists said that the predictions served as a second opinion (Textbox 1, Quote 5) and that the prediction tool was helpful for collecting arguments that supported the electrophysiologists when trying to “gather thoughts” about potential VT/VF occurrences (Textbox 1, Quote 6). Nevertheless, some electrophysiologists found that showing the probability score as a percentage with decimals created uncertainty, and the naming of parameters was sometimes found difficult to interpret (Textbox 1, Quote 7).

#### Is Visualization of Important Parameters Useful?

The prediction tool’s visualization of the most important parameters in the prediction of increased or decreased probability of VT/VF arrhythmia was found useful when the electrophysiologists agreed with the parameters presented. In patient cases 2 and 3, 6 (86%) and 5 (71%) of the electrophysiologists agreed that showing important parameters supported their decision-making. However, when the parameters represented poor data quality (Textbox 1, Quote 12), agreement was lower, for example, in patient Case 1 (43% agreed, 43% disagreed), or when the electrophysiologists were focused on predicting arrhythmias (Case 4 and Case 5) other than what the prediction tool was designed for (Textbox 1, Quote 13).

In general, presentation of important parameters provided explainability and supported decision-making by resembling the clinical interpretation process of what counts for or against the occurrence of VT/VF (Textbox 1, Quote 9). Several electrophysiologists found that visualization of important parameters created more confidence in the prediction tool than the probability score alone as the tool summed up many of the same assumptions that the electrophysiologists already had (Textbox 1, Quote 10). Listing the algorithm’s important parameters also enabled electrophysiologists to do in-situ validations of the prediction tool’s predictions by interpreting the data against the patient case (Textbox 1, Quote 11). However, in some cases, the electrophysiologists found that the parameters were based on wrong data from the ICD transmission. In those cases, it enabled electrophysiologists to check if the prediction tool based its predictions on wrong or poor data quality and to decide whether to trust the predictions or not (Textbox 1, Quote 12).

#### Does the Prediction Tool Reduce Time for Decision-Making?

The electrophysiologists found that the prediction tool could enable a reduction in time for decision-making in cases where they trusted the predictions. Moreover, 5 (71%) of the electrophysiologists agreed that the prediction tool can help reach a decision faster (Case 2 and Case 3). However, agreement was lower (29% in Case 1 and 57% in Case 5) when predictions were found to be uncertain or less useful for handling patients.

Several of the electrophysiologists expressed that once they become familiar with the system, they expect the AI tool will speed up decision-making and reduce the diagnostic workload. This indicates that establishing trust in AI predictions is essential. One of the electrophysiologists explained how time can be saved when personal trust in the prediction tool is developed (Textbox 1, Quote 14).

Across all cases, several electrophysiologists found that the probability score and the presentation of important parameters can reduce information search time. Typically, electrophysiologists must retrieve valuable information by clicking through multiple webpages in the ICD manufacturer’s web-based system, which the prediction tool summarizes in a table. Some electrophysiologists also speculated that the tool could support decision-making when patient input is inaccessible, such as when a patient does not answer the phone (Textbox 1, Quote 15). Other electrophysiologists considered that the tool can support workflow and reduce unnecessary time consumption for electrophysiologists by delegating decision-making to the technician (Textbox 1, Quote 16).

### Clinician Preimplementation Perspectives of the Prediction Tool: Acceptability, Adoption, Demand, and Implementation

#### Acceptability

Acceptability of the prediction tool was high when patient cases concerned VT/VF, as the risk predictions were found to be relevant. However, several electrophysiologists had expectations that the prediction tool would bring new and groundbreaking insights (Textbox 2, Quote 1) to support or challenge their decisions on which action to take. In cases where the task-technology fit was lower (Case 1, Case 4, and Case 5), acceptability was also lower (Textbox 2, Quote 2). For some of the electrophysiologists, the prediction tool was therefore considered “nice to have” rather than “need to have” (Textbox 2, Quote 3), while most of the electrophysiologists recognized the potential of the prediction tool. Some of the electrophysiologists considered the tool useful for standardizing decision-making across the electrophysiologist team by avoiding individual influences from recent experiences and thus achieving harmonization of individual treatment (Textbox 2, Quote 4).

#### Adoption

There was consensus that high precision is important for prediction tool adoption to happen. Several of the electrophysiologists emphasized that the positive or negative predictive value should be as unambiguous as possible, showing either low or high risk when the alarm is raised (Textbox 2, Quote 5). Other electrophysiologists emphasized that false positives or negatives hinder adoption, which they explained to be the case for the adoption of OptiVol (an early warning alarm for fluid-related decompensation). Here, the electrophysiologist team decided not to use it due to too many false positives (Textbox 2, Quote 6). Several electrophysiologists explained that acceptance and clinical adoption are collectively decided based on team experiences from real-world use (Textbox 2, Quote 7) and from experiencing that the prediction tool actually confirms decisions in everyday clinical practice (Textbox 2, Quote 8). Adoption can also be achieved through building trust in the tool by means of validation studies. Participants explained that trust is a precondition for adoption, which can be achieved by documenting effects in a randomized clinical trial and through algorithm validation in peer-reviewed journals (Textbox 2, Quote 9).

#### Demand

Several of the electrophysiologists emphasized that there is a high demand for workflow support in remote monitoring of cardiac device patients. They found the prediction tool useful for supporting more efficient prioritization and identification of important patient cases (Textbox 2, Quote 10). Others described the demand for screening support among the increasing number of nonspecialized hospitals where fewer electrophysiologists are at work. For example, the prediction tool could support technicians doing the initial prioritization work more effectively and efficiently; the prediction tool could decrease electrophysiologists’ patient information search time when handed over from technicians; and the prediction tool could function as “data help” by enabling junior doctors to get a form of senior help by consulting the tool (Textbox 2, Quote 11).

#### Implementation

To ensure successful implementation, some electrophysiologists described how remote monitoring clinics may want to be able to adjust the threshold of the prediction tool to fit local workflows and prioritization rules. For example, technicians and electrophysiologists should be able to configure the prediction tool and decide on related actions, such as “no need to take action” or “need to contact the patient.” Relatedly, several electrophysiologists explained that indications of low-risk patients are especially useful in supporting clinicians in handling low-risk transmissions (Textbox 2, Quote 12). Moreover, the electrophysiologists explained that the intention of using the prediction tool is dependent on easy access, as well as how well it presents data and alleviates the need for clicking through several web pages in remote monitoring systems (Textbox 2, Quote 13). Some electrophysiologists added that it is practical that the algorithm uses data already available in remote monitoring systems, which are used daily for decision-making in the clinic. Knowing the data creates transparency and enables in-situ validation of the correctness of the probability score, thereby increasing the likelihood for success with implementation of the prediction tool (Textbox 2, Quote 14).

**Themes, insights, and illustrative quotes describing clinicians’ preimplementation perspectives of the prediction tool.**

**Acceptability**

**Insights:** Expectations that the prediction tool would bring new and more groundbreaking insights; overall usefulness of the prediction tool is “nice to have;” clear purpose is decisive for acceptability; intension of use is tied to the prediction tool’s task-technology fit; harmonizing individual treatment; and avoiding being influenced by recent experiences and reducing individual bias.

- **Quote 1:*** […] it confirms the assessment you make, and that's fine, but it's not something groundbreaking, and that's okay too.* [Interview, Electrophysiologist #5]
- **Quote 2:*** […] I'm a little disappointed with the alarm, because for the cases I have looked at, the alarm has not given me much. […] if you had some cases with some ‘meat’ on such as a couple of treatment requiring VTs, I actually do think that getting a number, a risk score, will enable to better estimating the problem. I think that can be valuable […] I just think the cases were wrong. If you want to show that this algorithm gives value and then bring 2 cases with AF problems, which the algorithm does not handle, then that’s not optimal.* [Interview, Electrophysiologist #2]
- **Quote 3:*** It's always nice when something is supportive, I would say, but isn't it a “nice to have” and not a “need to have”?* [Interview, Electrophysiologist #4]
- **Quote 4:*** 20 years of experience or not. Perhaps, the advantage of the algorithm is that it is not influenced by what the individual clinician has experienced within the last month, and in this way helping to make more uniform conclusions.* [Interview, Electrophysiologist #5]

**Adoption**

**Insights:** High precision is important; false positives hinder adoption; using the prediction tool and getting confirmation in real life creates trust and enables adoption; the clinical team needs to decide on use; a randomized clinical trial is a precondition for acceptability; and algorithm validation supports trust.

- **Quote 5:*** If you want to come out with this, it must be something with a positive predictive value that is really good, so that you don’t get a lot of nonsense that you can’t use. The alarm should only be raised when there really is something.* [Interview, Electrophysiologist #3]
- **Quote 6:*** It needs to be easily accessible and we [team of electrophysiologists] have to agree that we trust it [the prediction tool]. We just have to say that yes it looks right. For example, the Optivol alarm had too many false positives, which gave a lot of extra work and everything, and we actually chose not to use it because there were too many sources of error, and you only really discover that when you work with it [new algorithms].* [Interview, Electrophysiologist #1]
- **Quote 7:*** I would say that it [prediction tool] would be an instrument that would have to be accepted in our group and then you would find it valuable when we all agree to take the red alarms first, and in that way use it to prioritize a bit.* [Interview, Electrophysiologist #5]
- **Quote 8:*** I just think I should see that it confirms our decisions in enough cases - then I would feel comfortable about colleagues leaning on it […] There is something about trying it out, you know how it is.* [Interview, Electrophysiologist #4]
- **Quote 9:*** Published studies of the algorithm would increase confidence yes, because then you know that someone with an understanding of making these models have said that it looks okay; someone externally who have validated it.* [Electrophysiologist #1]

**Demand**

**Insights:** Supporting better workflows; demand for prioritization and identification of important patient cases; increasing demand for screening tools in nonspecialist hospitals; demand for decreasing electrophysiologist’s information search time; supporting nurses and technicians to do prioritization work; and “data help” that enables junior doctors to get senior help.

- **Quote 10:*** When transmissions come in, it’s almost an unsorted list of transmissions […] The list is unprocessed, so with the algorithm it takes it a step further by nuancing what comes into CareLink [Medtronic’s remote monitoring dashboard] with some semi-quantitative markings. And, if it is reliable, then it would be valuable. Partly because you don’t overlook anything, and partly because you are confirmed that we must take these patients first, because we have experience that there can be trouble here.* [Interview, Electrophysiologist #5]
- **Quote 11:*** You could say that in this way, the young doctor can do without getting senior help by actually getting data help.* [Interview, Electrophysiologist #2]

 

**Implementation**

**Insights:** Demand for adjusting the threshold to local prioritization rules; clinics need to be able to configure the cutoff and threshold; making the prediction tool easily accessible and integrated in the list of transmissions supports the workflow; intention of use is dependent on easy access; and it is practical that the algorithm uses data that are already familiar to the clinicians.

- **Quote 12:*** Electrophysiologists don’t bother to hear about it if it is below a certain percentage […]. We have adopted some rules, e.g. if you have a patient and she has got a shock, and gets rare therapies and it goes over, then we don’t need to hear about it because we think there is not a big risk. You might well imagine that introducing this alarm will support handling low-risk transmissions.* [Interview, Electrophysiologist #3]
- **Quote 13:*** If it’s easily presented and you don’t have to go in and look through 4 pages and such and if it was on the front page and brought up “number of episodes” and information like that - if you could easily retrieve the information [from the prediction tool] or if it was printed on the list of transmissions we are working on, then it would also be a great help.* [Interview, Electrophysiologist #1]
- **Quote 14:*** What one would emphasize, is that the algorithm uses the same data that the clinician uses i.e. it’s the same data, just integrated according to a formula that clinicians do not currently have available.* [Interview, Electrophysiologist #5]

## Discussion

### Tackling the Sociotechnical Challenges of ML-Based Tools in Health Care

In bridging the sociotechnical gap between the development of ML-based tools and clinical implementation, this study explored the feasibility and clinical perspectives of using a prediction tool for improved workflows in ICD remote monitoring. We found that the feasibility of the ML-based tool is promising when the intended use of the tool is aligned with expectations, that is, by providing support for decision-making, visualizing useful information, and reducing time spent. The results also show that an actionable prediction tool is one that presents the reason for why the algorithm deemed as it did, such as in this study, by highlighting important data to be used for clinical evaluation and enabling clinicians to assess the algorithm’s outcome against their own evaluation [31,33].

However, the current prediction tool did not lead to change in clinical action, suggesting that ML and explainability techniques do not outperform specialized and experienced electrophysiologist evaluations, but at best confirm and support the interpretation of complex ICD device information along with a promise for a less time-consuming clinical workflow.

The contribution of this paper lies in the implications of the qualitative results suggesting that clinical end-users, clinical contexts, and workflows must be included throughout an overall iterative approach to design, development, and implementation. In the following sections, we will discuss the qualitative results concerning the sociotechnical challenges and implementation of ML-based tools for clinical decision support.

### Expectations Need to Align With the Intended Use of AI

In cases where misalignment emerged between the electrophysiologists’ expectations and intended use, the prediction tool was considered less useful and at best “nice to have” for clinical decision-making. For example, in cases where the ICD transmissions revolved around other types of arrhythmias than what the prediction tool was designed for and in cases where the electrophysiologists expected that the prediction tool should be capable of outperforming their own evaluation, disappointment was raised about the performance of the underlying AI algorithm. This aligns with recent studies that reported on physicians’ high expectations and attitudes toward medical AI [22,23,51,65]. The challenge of managing expectations has been addressed by a growing number of studies aimed at providing an explanation of algorithmic decisions at the time of inference [36] and by developing user interfaces with expectation adjustment techniques [66]. Recently, researchers focused on the early human-AI onboarding process of pathologists and found that presenting a global view of a prediction tool and its capabilities, limitations, and biases is key to the formation of initial impressions and appropriate mental models [67]. This suggests that the development of so-called explainability in the user interface is important, but communicating the intended use of the prediction tool is imperative for acceptance in the clinic. To achieve alignment of expectations, training programs for clinicians are critical when implementing medical AI tools.

### Trust Emerges From Real-World Use

Trust is another key factor for user acceptance and adoption of AI technologies. Trust is typically considered an issue in creating transparent and understandable algorithmic behavior, as opposed to seeing the prediction tool as a black box [55,68,69]. Extensive research on explainable AI and various approaches to achieve transparency have been suggested [11], yet experimental studies on whether these approaches achieve their intended effects in the real world are only just starting to emerge [38,39,69,70]. In this study, the electrophysiologists requested large-scale algorithm validation and prospective evaluations from clinical trials. However, an important observation was that trust in the prediction tool may only emerge from continuous use of the tool and from experiencing confirmation on individual evaluations in collaboration with the tool. There was general agreement among the electrophysiologists that visualization of the most important predictive parameters helped raise confidence and trust over time, and that adoption of the prediction tool would hinge on the collective decision among the team of electrophysiologists. Recent experimental studies have reported similar findings [69,71] and have demonstrated that adding an AI prediction tool to the clinical evaluation can increase clinician confidence [24]. The implication of understanding trust as emerging from real-world use is that when deploying medical AI in clinical settings, trust needs to be built bottom-up through weeks or months of trialing the new tool for clinicians to experience convincing reassurance. Therefore, initial implementation processes may benefit from simultaneous calibration and adaption of the tool to establish a human-AI partnership, and allowing the local team of clinicians to decide collectively how they choose to trust and use the tool in the clinic.

### Accuracy is Dependent on Workflow and Context

While AI algorithms have been validated and have been shown to have similar or higher accuracy than humans, recent studies of AI deployment in clinical settings report that professional autonomy, workflow, and local sociotechnical factors have impacts on how accuracy is perceived and used in clinical practice [24,43,45-47,50-54]. Bruun et al [24] found that overall performance was positively impacted among clinicians using an AI-prediction tool for assessing progression in early stage dementia and that clinicians’ professional autonomy impacts the use of medical AI in situated clinical practice. Additionally, the study by Beede et al [29] of a ML-based (deep learning) system used in clinics for the detection of diabetic eye disease indicated that several socioenvironmental factors, such as busy screening procedures, poor lighting conditions, and consideration of patient burden, have impacts on how AI accuracy is perceived in clinical screening practices. Similarly, we found that high accuracy becomes relative to the electrophysiologist’s evaluation of available information, the local circumstances, and the consequences that AI predictions have for taking action. For example, several electrophysiologists argued that AI prediction needs to be considered against patient-reported symptoms and that a full patient schedule may affect how the AI prediction is acted upon in practice. Moreover, in several cases, the electrophysiologists found the visualization of important parameters more useful than the prediction score itself. This indicates that AI accuracy needs to be understood as relational and dependent on available information and local workflows, which supports the vision of establishing a human-AI symbiosis that combines the predictive abilities of both the clinician and the AI prediction algorithm [32,33]. Finally, the wish for better visualization of data parameters over prediction accuracy indicates that the development of medical AI assistants needs to be carried out as close as possible to implementation in clinical practice with clinical end-users through iterative approaches [37,42,72] that can bridge the “AI chasm” [41] of scientifically sound algorithms and their use in meaningful real-world clinical applications.

### Limitations

The findings in this study are limited to the small number of study participants and patient cases. One electrophysiologist (PKJ) participated in co-design workshops, resulting in potential positive bias. Patient cases were selected to represent diversity in prediction capabilities, rather than the distribution in clinical practice, which may weaken the generalizability of the results. Only cases where the prediction tool provided true-positive and true-negative prediction outcomes were used, which means that the clinical feasibility of ML in cases with false-positive and false-negative outcomes [73] have not been explored. Future studies are needed to assess the implications of false prediction outcomes, as well as conduct algorithmic validation similar to recent related studies [14-21]. Limitations also involve data availability, that is, the data set used may entail algorithmic bias [13] and the study participants may have been more positive toward innovative AI technology since all of the study participants were from a tertiary university hospital and constituted a rather homogenous group of highly specialized physicians. The AI studied has limitations, because only the random forest ML-based algorithm was evaluated with electrophysiologists. These types of methods are commonly used in medical applications [21,74,75] because of their high classification accuracy and capabilities for handling data with imbalanced classes [50] while providing easily accessible, if limited, global intelligibility through the visualization and ranking of parameter importance [55]. This work will benefit from being validated in a large-scale multicenter study with higher diversity in participating electrophysiologists and workflows. It will be imperative to conduct prospective clinical trials evaluating the algorithm against standard care with regard to workload, cost-effectiveness, and hard clinical endpoints.

### Conclusions

This study shows that a tool based on ML for the prediction of VT/VF in remote monitoring of ICD patients has the potential to support electrophysiologists’ decision-making. While the prediction tool was regarded as “nice to have” rather than “need to have” in its current form, the tool demonstrated potential for supporting clinical decision-making, as it provided reassurance, increased confidence, and indicated the potential for reducing information search time, as well as enabled delegation of decisions to nurses and technicians. The findings also indicate that trust in the prediction tool, acceptable data quality, and clearly defined intended use are decisive for end-user acceptance and that adoption hinges on successful clinical implementation. This suggests that clinical end-users’ sociotechnical contexts and workflows need to be taken into consideration early on and continuously throughout a participatory design process to address the sociotechnical gap between the development and implementation of medical AI in cardiac care.

## Appendix

Multimedia Appendix 1 Questionnaire used before the electrophysiologists are presented with the prediction tool results.

Multimedia Appendix 2 Questionnaire after the electrophysiologists have received the prediction tool results.

Multimedia Appendix 3 The semistructured interview guide.

## Abbreviations

AI: artificial intelligence
ICD: implantable cardioverter defibrillator
LSTM: long short-term memory
ML: machine learning
VF: ventricular fibrillation
VT: ventricular tachycardia
