# Supplementary material for: Clinician Preimplementation Perspectives of a Decision-Support Tool for the Prediction of Cardiac Arrhythmia Based on Machine Learning: Near-Live Feasibility and Qualitative Study
Source: JMIR Hum Factors. 2021 Nov 26;8(4):e26964. doi: 10.2196/26964 (PMC8665383; doi:10.2196/26964)
Supplement: Multimedia Appendix 1 [file humanfactors_v8i4e26964_app1.pdf]

Feasibility study

* Required

1. Please write your initials *

2. Please write the number of the case you are working on *

Questionnaire I: Based on the given case information

3. What action will you take?

Mark only one oval per row.

	Meget uenig	Uenig	Hverken uenig eller enig	Enig	Meget enig
I want to contact the patient					
I want to request/re-schedule procedure					
I will not take any action					
I miss information					
I want to do something else					

4. Other/comments
