# Supplementary material for: Clinician Preimplementation Perspectives of a Decision-Support Tool for the Prediction of Cardiac Arrhythmia Based on Machine Learning: Near-Live Feasibility and Qualitative Study
Source: JMIR Hum Factors. 2021 Nov 26;8(4):e26964. doi: 10.2196/26964 (PMC8665383; doi:10.2196/26964)
Supplement: Multimedia Appendix 2 [file humanfactors_v8i4e26964_app2.pdf]

Appendix B

Questionnaire II: After seeing the AI-tool results

5. What action will you take after you have seen the results of the AI-tool?

Mark only one oval per row.

	Meget uenig	Uenig	Hverken uenig eller enig	Enig	Meget enig
I want to contact the patient					
I want to request/re-schedule procedure					
I will not take any action					
I miss information					
I want to do something else					

6. I want to change my action

Mark only one oval.

- Yes
- No

7. Other/comments

8. Decision

Mark only one oval per row.

	Meget uenig	Uenig	Hverken uenig eller enig	Enig	Meget enig
The AI-tool supported my decision-making in this case					
Visualizing important parameters supported my decision-making in this case					
The AI-tool could help me reduce time for decision in this case					

This content is neither created nor endorsed by Google.

Google Forms
