# Supplementary material for: Clinician Preimplementation Perspectives of a Decision-Support Tool for the Prediction of Cardiac Arrhythmia Based on Machine Learning: Near-Live Feasibility and Qualitative Study
Source: JMIR Hum Factors. 2021 Nov 26;8(4):e26964. doi: 10.2196/26964 (PMC8665383; doi:10.2196/26964)
Supplement: Multimedia Appendix 3 [file humanfactors_v8i4e26964_app3.pdf]

Final Interview Questions

1) What do you think about the alarm?
- How does the alarm affect your decision making? [Acceptability]

2) Do you want to use the alarm if it became available e.g. in CareLink? [Intent of continued use]

3) Do you trust the alarm? [Trust]
- What is important to increase confidence in the alarm? [Adaptability]

4) The alarm shows data parameters - how useful is it? [Interpretability]
- Is it important that the parameters are displayed in an understandable way?

5) The alarm shows the probability in % - how useful is it? [Interpretability]

6) Would you recommend other clinicians to use it? [Demand]
- Is there a difference in the need among younger vs. experienced doctors?
- Will the pace technicians be able to use the alarm?
- How do you assess the need for the alarm in remote monitoring? [Demand]

7) What does it take for the alarm to be used in Paceamb? [practicality + implementation]
- Will it require reorganization / something extra? [Adaptation + Expansion]

8) Do you think the alarm will be able to reduce readmissions?

9) Can we use audio/video data for teaching or conferences?

10) Do you have any concluding comments? Has testing the alarm changed your attitude towards AI methods?
